# Single-cell RNA-seq reveals the diversity of trophoblast subtypes and patterns of differentiation in the human placenta 

**DOI:** 10.1038/s41422-018-0066-y

**Published:** 2018-07-24

**Authors:** Yawei Liu, Xiaoying Fan, Rui Wang, Xiaoyin Lu, Yan-Li Dang, Huiying Wang, Hai-Yan Lin, Cheng Zhu, Hao Ge, James C. Cross, Hongmei Wang

**Affiliations:** 1 State Key Laboratory of Stem Cell and Reproductive Biology, Institute of Zoology, Chinese Academy of Sciences, 100101 Beijing, China; 2 Biomedical Pioneering Innovation Center, College of Life Science, Peking University, 100871 Beijing, China; 3 Department of Experimental Radiation Oncology, The University of Texas MD Anderson Cancer Center, Houston, TX 77030 USA; 4 Department of Obstetrics and Gynecology, The 306th Hospital of PLA, 100101 Beijing, China; 5 Department of Obstetrics and Gynecology, Beijing Shijitan Hospital, 100038 Beijing, China; 6 Departments of Biochemistry and Molecular Biology, Comparative Biology and Experimental Medicine, Obstetrics and Gynecology, and Medical Genetics, University of Calgary, Calgary, AB T2N 4N1 Canada

## Abstract

The placenta is crucial for a successful pregnancy and the health of both the fetus and the pregnant woman. However, how the human trophoblast lineage is regulated, including the categorization of the placental cell subtypes is poorly understood. Here we performed single-cell RNA sequencing (RNA-seq) on sorted placental cells from first- and second-trimester human placentas. New subtypes of cells of the known cytotrophoblast cells (CTBs), extravillous trophoblast cells (EVTs), Hofbauer cells, and mesenchymal stromal cells were identified and cell-type-specific gene signatures were defined. Functionally, this study revealed many previously unknown functions of the human placenta. Notably, 102 polypeptide hormone genes were found to be expressed by various subtypes of placental cells, which suggests a complex and significant role of these hormones in regulating fetal growth and adaptations of maternal physiology to pregnancy. These results document human placental trophoblast differentiation at single-cell resolution and thus advance our understanding of human placentation during the early stage of pregnancy.

## Introduction

The first cell fate decision during human embryo development divides the embryonic cells into two lineages, i.e., the inner cell mass (ICM) and the trophectoderm, which further develop into the embryo proper and the main part of the placenta, respectively.^1^ The placenta is a transient organ that is essential for anchoring the conceptus, preventing its rejection by the maternal immune system, and transporting nutrients and waste between the fetus and the mother.^2^ The placenta performs these functions via multiple specialized cell types that result from coordinated genetic, epigenetic and physiological regulation during human placentation. Any dysregulation in placentation may lead to poor pregnancy outcomes, such as miscarriage, intrauterine growth restriction and preeclampsia, and can affect the lifelong health of both the mother and the fetus.^3–5^

The villus is the functional unit of the placenta and consists of an outer epithelial trophoblast layer and a stromal cell core, derived from the trophectoderm and the extraembryonic mesoderm, respectively.^6^ The stromal cell core contains fetal endothelial cells, mesenchymal stromal cells (MSCs), Hofbauer cells^7^ amongst others. MSCs in the human placenta have been reported to be fibroblast-like cells with differentiation capabilities and immunomodulatory properties.^8^ Hofbauer cells are fetal macrophages that may be involved in the phagocytosis of cellular debris and the modulation of human placental development by enhancing villous branching.^9,10^

The mature human placenta is described as having three main types of epithelial trophoblasts: cytotrophoblasts (CTBs), the syncytiotrophoblast (STB) and extravillous trophoblasts (EVTs). CTBs form a single layer that lines the stromal cell core and serves as the source of the replenishment of the STB and EVTs.^6,11^ EVTs are differentiated trophoblast cells that migrate from the tips of the placental villi, proliferate and differentiate to form a trophoblast cell column.^12^ EVTs at the distal region of the column then detach from the villi and invade the interstitial compartments of the maternal uterine wall, thereby anchoring the fetus and remodeling the uterine spiral artery to facilitate fetal-maternal nutrient transfer.^2,13–15^ The STB is a multinucleated structure that covers the entire surface of the villous tree throughout pregnancy. It contains approximately 58 billion nuclei and has a surface area of 12–14 square meters at term.^16^ The maintenance of a functional STB depends on the shedding of apoptotic nuclei and cytoplasm from the STB surface into the maternal circulation and the continuous incorporation of new cell components via the fusion of CTBs from the CTB layer underneath the STB.^17,18^

Although placental cells have been traditionally classified as described above, the extent to which it is useful to define subtypes of trophoblast cells and stromal cells and the relationships between cell subtypes and functions remain unclear. Single-cell RNA-seq has been a powerful tool for the identification of cell subtypes in different tissues.^19,20^ Two studies have examined the human placental transcriptome from later stages: Pavlicev et al. explored 87 single-cell transcriptomes from the human placenta at term and investigated the cell-cell interactome between the fetal trophoblast and the maternal endometrial stromal cells;^21^ Tsang et al. studied 24000 nonmarker selected cells from full-term normal placentas and second trimester preeclamptic placentas and re-confirmed the differentiation relationships between the three known populations, i.e., CTBs, EVTs and the STB.^22^ Both studies used placental tissues from second and third trimester pregnancies, and their endpoints were not the identification of new populations of cells in the placenta.

In the present study, we isolated human villous stromal cells (STRs), CTBs, the STB, and EVTs during the first and second trimesters of pregnancy and monitored the transcriptome dynamics of 1567 cells at single-cell resolution. We identified 14 subtypes of placental cells and characterized their functions using bioinformatics analyses and immunostaining verifications. Amongst our unexpected findings is that 102 polypeptide hormone genes were expressed by various subtypes of placental cells. Our study builds a strong foundation for understanding how the human placenta develops and functions to maintain a healthy pregnancy.

## Results

### Isolation and validation of the placental cells used for single-cell RNA-seq

To identify possible new subtypes of placental cells and characterize their unique transcriptional signatures in the developing human placenta, a nonmarker-selected strategy should be the first choice to collect the starting cells (CTBs, the STB, EVTs, villous STRs, etc.). However, because the proportions of the different types of known placental cells are so different in the placenta, an unbiased selection strategy would probably miss the potentially important but rare populations. We chose not to use fluorescence-activated cell sorting (FACS) technology because the primary cells, especially placenta-derived trophoblast cells, are very vulnerable to damage and the high speeds experienced by cells during FACS can be detrimental (data not shown). In contrast, magnetic-activated cell sorting (MACS) technology is beneficial for maintaining better cell vitality. The potential purity issue associated with this technology can be overcome by unsupervised clustering analysis.^23^ We specifically chose healthy placental tissues from first and second trimester pregnancies because placental cellular lineage commitment occurs mainly during these stages.

**Fig. 1:**
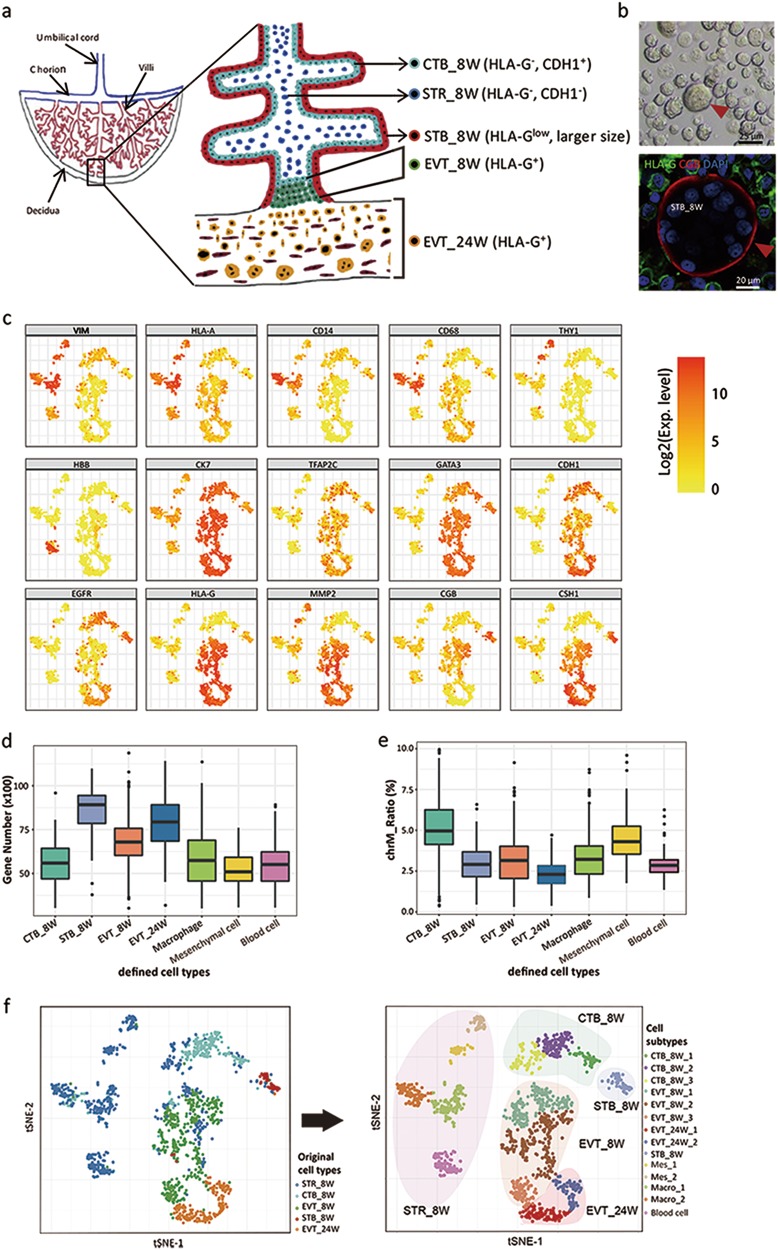
Isolation and validation of placental cells from 8 W and 24 W human placentas for single-cell RNA-seq. **a** Diagram of the human placental villi showing the localization of each cell type involved in this study and the markers used to sort these cells. CTB_8W, STR_8W, STB_8W, and EVT_8W: cytotrophoblasts, stromal cells, syncytiotrophoblast and extravillous trophoblasts sorted from the 8 week placental villi, respectively; EVT_24W, extravillous trophoblasts sorted from 24 week decidua. **b** Representative images showing bright field (upper panel) and immunofluorescence staining of STBs (bottom panel; red arrowhead). The beta subunit of chorionic gonadotropin (CGB) is a canonical marker for the STB. DAPI staining shows the nuclei (here and after). **c** Dot plot showing the expression of canonical marker genes for the defined cell types. **d** Box plot of the gene numbers detected in the defined cell types. **e** Box plot showing the distribution of the ratio of reads mapping to mitochondria in each cell type. **f** T-SNE distributions of single cells from the 7 defined cell types sorted by MACS from the 8 W and 24 W placentas

To sort the different placental cells via MACS, we selected human leukocyte antigen-G (HLA-G) and cadherin 1 (CDH1) as surface markers. The specificities of the antibodies were verified by immunofluorescence staining (Supplementary information, Figure S1a). HLA-G, a marker of EVTs,^24^ was highly and specifically expressed in EVTs, slightly expressed in the STB, and could not be detected in the CTBs and STRs. In contrast, CDH1, which is a marker of epithelial cells,^24^ was highly expressed in the CTBs and some EVTs but not in the STB or STRs (Supplementary information, Figure S1a). After confirming the efficacies of the antibodies, we enzymatically digested human placental villi at 8 weeks and decidua at 24 weeks of gestation and subjected them to MACS (Supplementary information, Figure S1b, c). Four populations of cells from the villi at 8 weeks of pregnancy and one population from the decidua at 24 weeks of pregnancy were harvested. We named these five populations of cells as EVT_8W (HLA-G^+^, mononucleated, smaller size), STB_8W (HLA-G^low^, multinucleated, larger size), CTB_8W (CDH1^+^ and HLA-G^-^), STR_8W (HLA-G^-^ and CDH1^−^), and EVT_24W (HLA-G^+^) (Fig. 1a). The STB_8W were multinucleated, having 5–15 nuclei, and were 25–80 μm in diameter in the trypsin-digested suspension of the first trimester villi (Fig. 1b). As summarized in Supplementary information, Figure S1d, a total of 1567 single cells were collected from 8 embryos. After filtering out the low-quality cells (see Materials and methods), 1471 cells were selected for further analysis.

### Single-cell transcriptome profiles distinguished 14 discrete subtypes of placental cells

As presented in Supplementary information, Figure S2a, b, distinct cell types from different embryos could cluster together based on both principal component analysis (PCA) and the spatially mapped t-distributed stochastic neighbor embedding (t-SNE) analyses, which indicated a negligible batch effect in our study. The purity of the cells based on MACS was lower than that based on FACS, and we calculated the true cell rates, false negative cell rates and false positive cell rates in the four cell populations based on the marker genes expressed in each cell from the 8 week human placenta (Supplementary information, Figure S2c). Unsupervised clustering analysis using t-SNE method revealed the unbiased distribution of the 1471 cells. Comparisons of the expression levels of well-characterized marker genes subsequently categorized these cells into 7 populations (Fig. 1c). As presented in Fig. 1c, the clusters exhibiting high expression of VIM and HLA-A were cells from the stromal cell core. Among these cells, the CD14- and CD68-positive clusters were macrophages (Hofbauer cells), and the clusters with high expression of THY1 were mesenchymal stromal cells. The cluster with high expression of HBB was blood cells. The cell clusters with high expression of CK7 were those of trophoblast origin, and these cells also exhibited high expression of TFAP2C and GATA3, which are the two generic trophoblast markers.^24^ The CDH1 and EGFR double-positive and HLA-G-negative clusters were CTB_8W cells. The cell clusters with high expression of *HLA-G* and *MMP2* [27], which are marker genes identifying EVTs, were EVT_8W and EVT_24W cells. STB_8W cells exhibited high expression of *CGB* and *CSH1*, genes used to recognize the STB.^24^ Collectively, 7 populations of human placental cells were identified, i.e., CTB_8W, EVT_8W, STB_8W, macrophages, mesenchymal stromal cells, blood cells from the 8 W human placenta and EVT_24W cells from the decidua of 24 W human placenta. The numbers of genes expressed for these 7 types of cells ranged from 4,000 to 10,000, and the different types of cells exhibited distinct numbers of expressed genes (Fig. 1d). The ratio of mapping reads to mitochondrial genes was relatively low, indicating that these cells exhibited high vitality (Fig. 1e and Supplementary information, Figure S2d). To further characterize the molecular characteristics of these 7 types of cells, we identified genes that were specifically expressed at high levels in each cell type. Using these genes, we performed functional annotation by gene ontology (GO) analysis; the highly enriched terms are presented in Supplementary information, Figure S2e. For example, the enrichment of GO terms in CTB_8W cell transcriptomes suggests these cells govern anion and vitamin transport. The transcriptomes of the EVT_8W cells indicate these cells control organic anion transport and epithelial cell proliferation. In accordance with the invasive property of the EVT_24W cells, the GO terms enriched in these cells were associated with extracellular matrix organization and cellular component movement. The enriched terms for the STB_8W cells were associated with glycoprotein hormones and small molecule transport, which accords with the secretory and transport functions of the syncytiotrophoblast (Supplementary information, Figure S2e).

Further investigation revealed subtypes in some of these cell populations on the t-SNE plot (Fig. 1f). For example, the CD14-positive macrophages comprised two subtypes, which we termed Macro_1 and Macro_2. The CD90-positive mesenchymal stromal cells also fell into 2 subtypes, i.e., Mes_1 and Mes_2. The CTB_8W cells were scattered into three groups that we named CTB_8W_1, CTB_8W_2 and CTB_8W_3. The EVT_8W cells also scattered into three groups that were designated EVT_8W_1, EVT_8W_2 and EVT_8W_3. Two subtypes were found in EVT_24W, i.e., EVT_24W_1 and EVT_24W_2 cells (Fig. 1f). In total, 14 subtypes of placental cells were identified.

### Three CTB subtypes present in the first trimester placenta

**Fig. 2:**
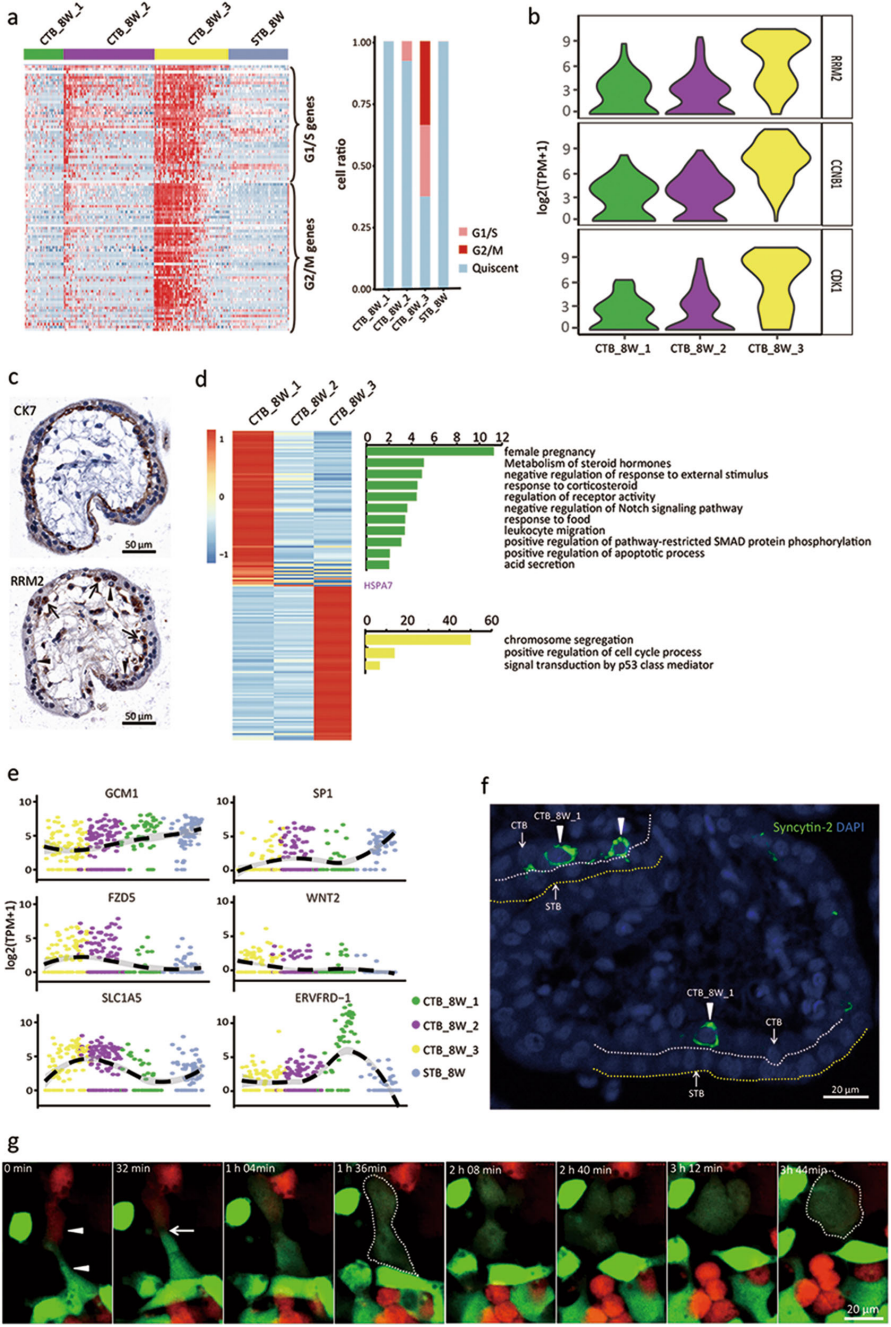
Three CTB subtypes were identified in the first trimester human placenta. **a** Heatmap showing the expression of cell cycle-related genes in the 3 different CTB subtypes. Cells expressing over 100 cell cycle-related genes were defined as cycling cells. **b** Violin plot displaying the cell cycle-related marker genes for CTB_8W cells. **c** Immunohistochemistry staining of human first trimester placental villi using the indicated antibodies. The arrows indicate RRM2-positive CTB cells, and the arrowheads indicate RRM2-negative CTB cells. **d** GO analysis of the DEGs for the three different subtypes of CTB_8W cells. **e** Dot plot showing the expression of the indicated genes in the indicated subtypes of cells. **f** Localization of Syncytin-2 in human placental villi from 8 weeks of pregnancy by immunofluorescence staining. The white arrowheads indicate CTB_8W_1 cells. DAPI staining shows the nuclei. STB, syncytiotrophoblast layer (the area between white and yellow dotted lines); CTB, cytotrophoblast cells. **g** Representative still images corresponding to Supplementary information, movie S1, time-lapse imaging of the fusion process of 293 T cells mediated by Syncytin-2. The arrowheads indicate two single cells (transfected with Syncytin-2, together with either EGFP or mCherry) about to undergo fusion. The arrow indicates the fusion site. The circled dashed lines indicate the fused cells

Cell cycle analysis indicated that among the three subtypes of CTB_8W cells (Fig. 1f), the CTB_8W_3 cells exhibited the highest proliferative activity (Fig. 2a). Violin plots revealed that many of the genes that were highly expressed in the CTB_8W_3 cells were cell cycle-related genes, including *RRM2*, *CCNB1* and *CDK1* (Fig. 2b). Using the RRM2 antibody, we localized the CTB_8W_3 cells in the placental villi by immunohistochemistry staining (Fig. 2c). GO analysis using the differentially expressed genes (DEGs) illustrated that the CTB_8W_3 cell-enriched genes were associated with positive regulation of the cell cycle process and chromosome segregation (Fig. 2d).

The CTB_8W_1 cells exhibited transcriptomes suggesting the least proliferative capacity (Fig. 2a). In our previous work, we found that not all CTBs are competent for fusion. Only CTBs that have exited the cell cycle can fuse.^25^ We believed that CTB_8W_1 cells might be the fusion-competent cells among the CTBs. To test this hypothesis, we first compared the expression of potentially important fusion genes, including *ERVFRD-1* (*Syncytin-2*), *GCM1*, *SLC1A5*, *SP1*, *FZD5*, and *WNT2*,^26–28^ in the subtypes of CTB_8W cells and STB_8W cells (Fig. 2e). Among all the genes examined, *Syncytin-2*, which encodes a membrane-localized fusogenic protein in mammals that directly mediates cell-cell fusion,^29^ exhibited a very high expression level in CTB_8W_1 cells compared with the other two CTB subtypes. We performed immunofluorescence staining on the 8 week placental villi using anti-Syncytin-2 antibody and localized the CTB_8W_1 cells in the CTB layer (Fig. 2f). To test whether Syncytin-2 was sufficient to induce cell fusion, two populations of non-fusing 293T cells were transfected with Syncytin-2 together with either enhanced green fluorescent protein (EGFP) or mCherry. The two groups of cells were mixed and subjected to time-lapse live-cell microscopy. As presented in Fig. 2g and Supplementary information, Movie S1, the green and red fluorescent cytoplasm flowed between the two cell types and the newly formed syncytium exhibited homogeneously mixed double-fluorescent cytoplasm, suggesting that Syncytin-2 is sufficient to induce the fusion of human cells. Moreover, the expression of Syncytin-2 in one cell was sufficient to induce its fusion with a cell that did not express Syncytin-2 (Supplementary information, Movie S2 and Figure S3a). These observations, together with the high level of Syncytin-2 expression in the CTB_8W_1 cells, strongly suggest that the Syncytin-2-positive CTB_8W_1 cells were fusion-competent trophoblast cells. Due to the critical roles of transcription factors (TFs) in cell lineage determination,^30^ we performed MAster Regulator INference algorithm (MARINa) analysis to elucidate the top-ranked TFs controlling trophoblast fusion (Supplementary information, Table S2 and Figure S3b). Their potential involvement in trophoblast fusion needs to be further explored. We also examined the main signaling pathways involved in trophoblast cell fusion by Gene Set Enrichment Analysis (GSEA) using the database from the Kyoto Encyclopedia of Genes and Genomes (KEGG), and the top-ranked signaling pathways are illustrated in Supplementary information, Figure S3c. The enriched terms in the CTB_8W_1 cells based on the GO analysis were associated with pregnancy and the metabolism of steroid hormones (Fig. 2d).

The CTB_8W_2 cell subtype also exited the cell cycle and did not express Syncytin-2. The function of these cells remains to be further determined.

### Different EVT subtypes imply a possible differentiation route during the first and second trimesters of pregnancy

**Fig. 3:**
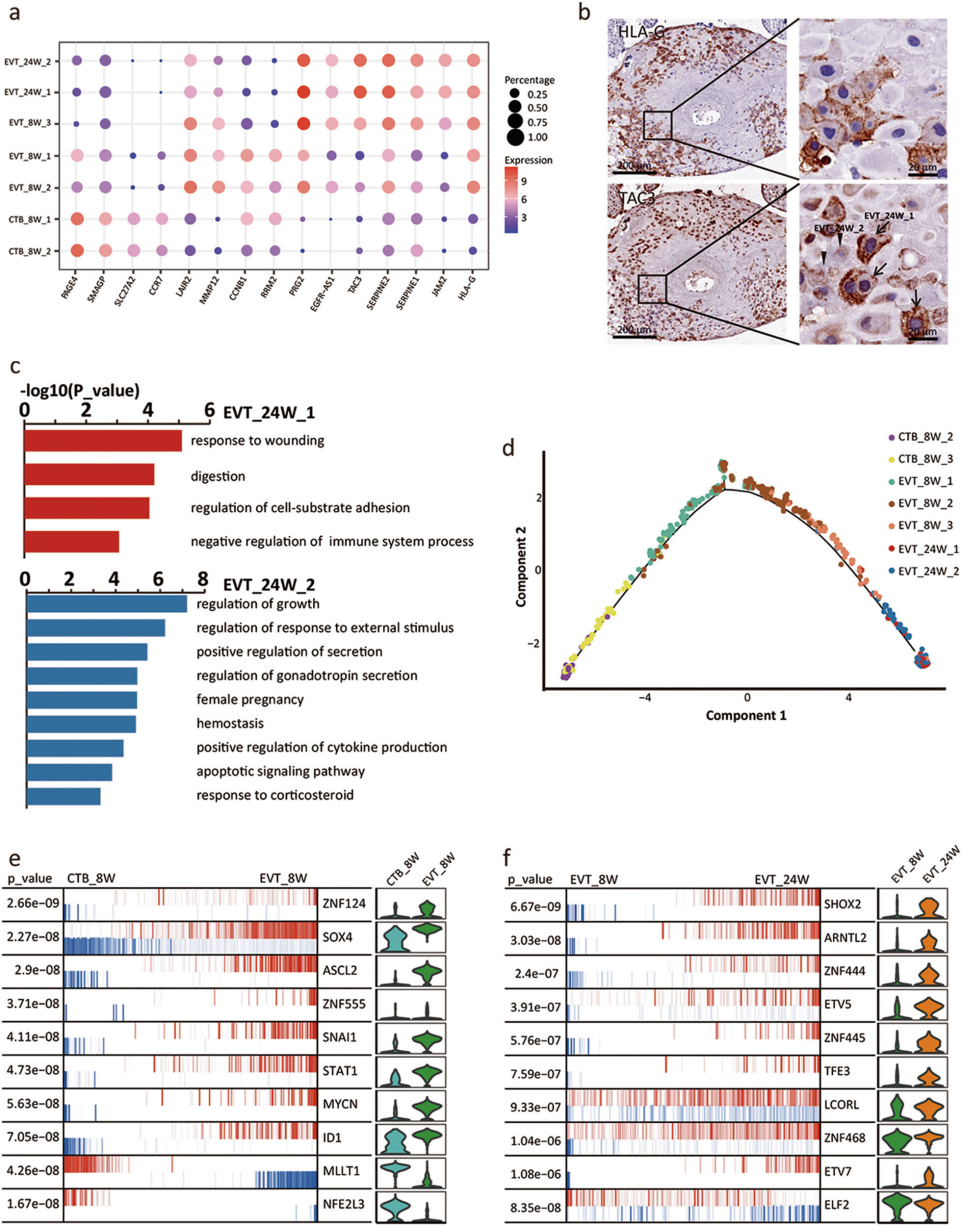
EVT subtypes and differentiation of CTBs into EVTs in the first and second trimesters. **a** Dot plot showing candidate marker genes for the indicated cell subtypes. **b** Immunohistochemistry staining of HLA-G and TAC3 in the decidua of 24 W human placenta. Arrows, EVTs with high expression of TAC3; arrowheads, EVTs with low expression of TAC3. **c** GO analysis of two subtypes of EVT_24W cells. **d** Pseudotime analysis of the indicated cells. Component 2 displays the differentiation of the CTB and EVT cells over time. **e, f** Top 10 transcription factors regulating the differentiation from CTB_8W to EVT_8W, and from EVT_8W to EVT_24W cells. Blue and red, the repressed and activated targets for each TF, respectively

Different EVT subtypes from both 8 week human placenta and 24 week decidua were verified. The differentially expressed genes for each subtype of cells are listed in Supplementary information, Table S1 and presented in a dot plot (Fig. 3a). The EVT_8W cells clustered into three populations that we named EVT_8W_1, EVT_8W_2, and EVT_8W_3. GO analysis revealed that the major terms enriched in EVT_8W_1 were associated with the cell cycle and cell division, suggesting that this cell subtype might have proliferative potential. RRM2, which plays an important role in DNA replication, was found to be highly expressed in EVT_8W_1 cells compared with the other two EVT_8W subtypes. GO analysis revealed that the enriched terms for EVT_8W_3 cells were associated with receptor activity regulation and the immune response (Supplementary information, Figure S4a), and EVT_8W_3 cells displayed a strong similarity with EVT_24W cells in terms of gene expression. For example, both of these subtypes exhibited high expression of Tachykinin-3 (TAC3), Plasminogen activator inhibitor-1 (SERPINE1), PRG2 and JAM2 (Fig. 3a). EVT_8W_2 cells exhibited moderate expression levels of the marker genes of the other 2 cell subtypes (Fig. 3a). To validate the existence of these cell subtypes, we performed immunohistochemistry staining with anti-RRM2 and anti-SERPINE1 antibodies on serial sections of paraffin embedded human placental villi. Our results revealed that RRM2-positive EVTs were localized at the proximal end of the cell column, and the EVTs exhibited little or low expression of RRM2 at the distal end of the column (Supplementary information, Figure S4b). There were two subtypes in the distal EVTs, i.e., SERPINE1-positive and SERPINE1-negative (Supplementary information, Figure S4b).

We also found EVT_24W cells clustered into 2 subtypes, which we named EVT_24W_1 and EVT_24W_2. The differentially expressed genes were analyzed and are listed in Supplementary information, Table S1. We selected TAC3 as a marker to distinguish these two subtypes on serial sections of paraffin-embedded 24 week decidua (Fig. 3a), and they could be clearly separated by immunohistochemistry staining (Fig. 3b). GO analysis of the enriched genes revealed that they were associated with the response to wounding, digestion and the negative regulation of the immune system in the EVT_24W_1 cell subtype whereas the enriched terms in EVT_24W_2 cells were associated with growth regulation, gonadotropin secretion and pregnancy (Fig. 3c).

EVTs originate from CTBs. To elucidate the possible process of differentiation from CTBs into EVTs, we performed a pseudotime analysis on CTB_8W, EVT_8W and EVT_24W cells. This analysis predicted a well-ordered differentiation pattern from CTB_8W_2 cells to CTB_8W_3 cells and then to EVT_8W (from EVT_8W_1 cells to EVT_8W_3 cells) and EVT_24W cells (Fig. 3d). We next performed MARINa and GSEA to infer the potential TFs and signaling pathways that might control the possible cell subtype transitions. Among these, SNAI1, STAT1, ASCL2 and ID1 have been reported to play an essential role in EVT cell differentiation and the maintenance of EVT cell characteristics^31–33^ whereas the functions of the other TFs require further exploration (Fig. 3e, f and Supplementary information, Figure S4c and Table S2). We also found that classical signaling pathways, such as JAK_STAT and MAPK pathways, might be involved in the process of differentiation from CTB to EVT cells (Supplementary information, Figure S4c).

### Identification of new subtypes of macrophages and mesenchymal stromal cells from the first trimester villous stromal core

**Fig. 4:**
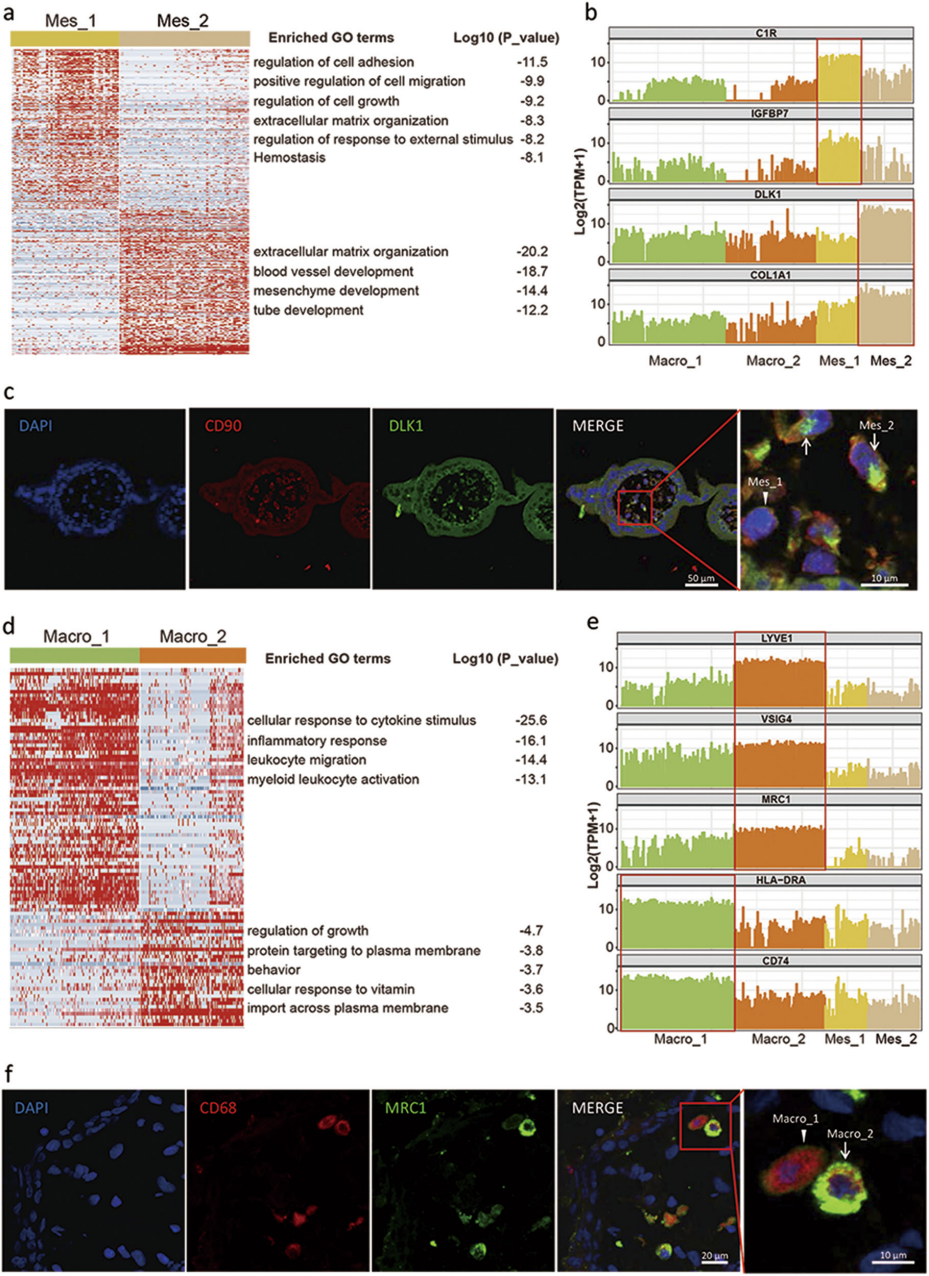
Identification of new subtypes of macrophages and mesenchymal stromal cells from the first trimester villous stromal core. **a** GO analysis of two subtypes of mesenchymal stromal cells. **b** Line plot showing the candidate marker genes for Mes_1 and Mes_2 cells. **c** Immunofluorescence staining of the indicated molecules in the 8 week human placental villi. The white arrows and arrowheads indicate mesenchymal stromal cells with high or low expression of DLK1, respectively. **d** GO analysis of two subtypes of macrophages. **e** Line plot showing the candidate marker genes for Macro_1 and Macro_2. **f** Immunofluorescence staining of the indicated molecules in the 8 week human placental villi. The white arrow and arrowhead indicate macrophages with high and low expression of MRC1, respectively

We were able to verify the presence of mesenchymal stromal cells and Hofbauer cells based on validated markers (Fig. 1c). Further analysis revealed that the CD90, ENG and CD74 tri-positive mesenchymal stromal cells were clustered into two subtypes, i.e., Mes_1 and Mes_2 cells. Based on GO analysis using the DEGs between the two subtypes, it appears from the highly enriched terms that Mes_1 cells participate in the regulation of cell adhesion and migration, whereas enriched terms in the Mes_2 cells indicate involvement in the development of blood vessels, the mesenchyme and the tube (Fig. 4a). Using an antibody against DLK1, a molecule that is highly expressed in Mes_2 cells (Fig. 4b), we successfully separated the Mes_2 and Mes_1 cells in the stromal core of the 8 week human placental villi using immunofluorescence (Fig. 4c). We also isolated CD90-positive mesenchymal stromal cells from the 8 week human placenta using FACS, and found by immunofluorescence that there were two distinct subtypes with high and low expression of DLK1 (Supplementary information, Figure S5a).

The CD68-positive Hofbauer cells also clustered into two subtypes, designated Macro_1 and Macro_2 (Fig. 1f). Compared with Macro_2 cells, Macro_1 cells seemed to be in an activated state and highly expressed genes encoding different chains of the HLA class II histocompatibility antigen that have been demonstrated to be involved in the endocytic route of antigen presentation (Supplementary information, Table S1). Enzymes, such as CTSS and LYZ, which are involved in this process were also highly expressed in this cell subtype. The enriched terms for Macro_1 cells included genes related to cytokine stimuli, the inflammatory response and myeloid leukocyte activation (Fig. 4d). This subtype of activated macrophages might be implicated in the removal of dead cells or cellular debris during the early development of the human placenta. When we used immunofluorescence to detect CD74 and MRC1, markers for Macro_1 and Macro_2 cells, respectively (Fig. 4e), we could detect these two subtypes of cells among the CD68-positive macrophages with anti-MRC1 antibody in human placental villi in vitro (Fig. 4f). We also isolated CD68-positive macrophages and found that presence of CD74 could separate these cells into two subtypes in vitro (Supplementary information, Figure S5b).

### Epigenetic modification in the determination of trophoblast cell fate

**Fig. 5:**
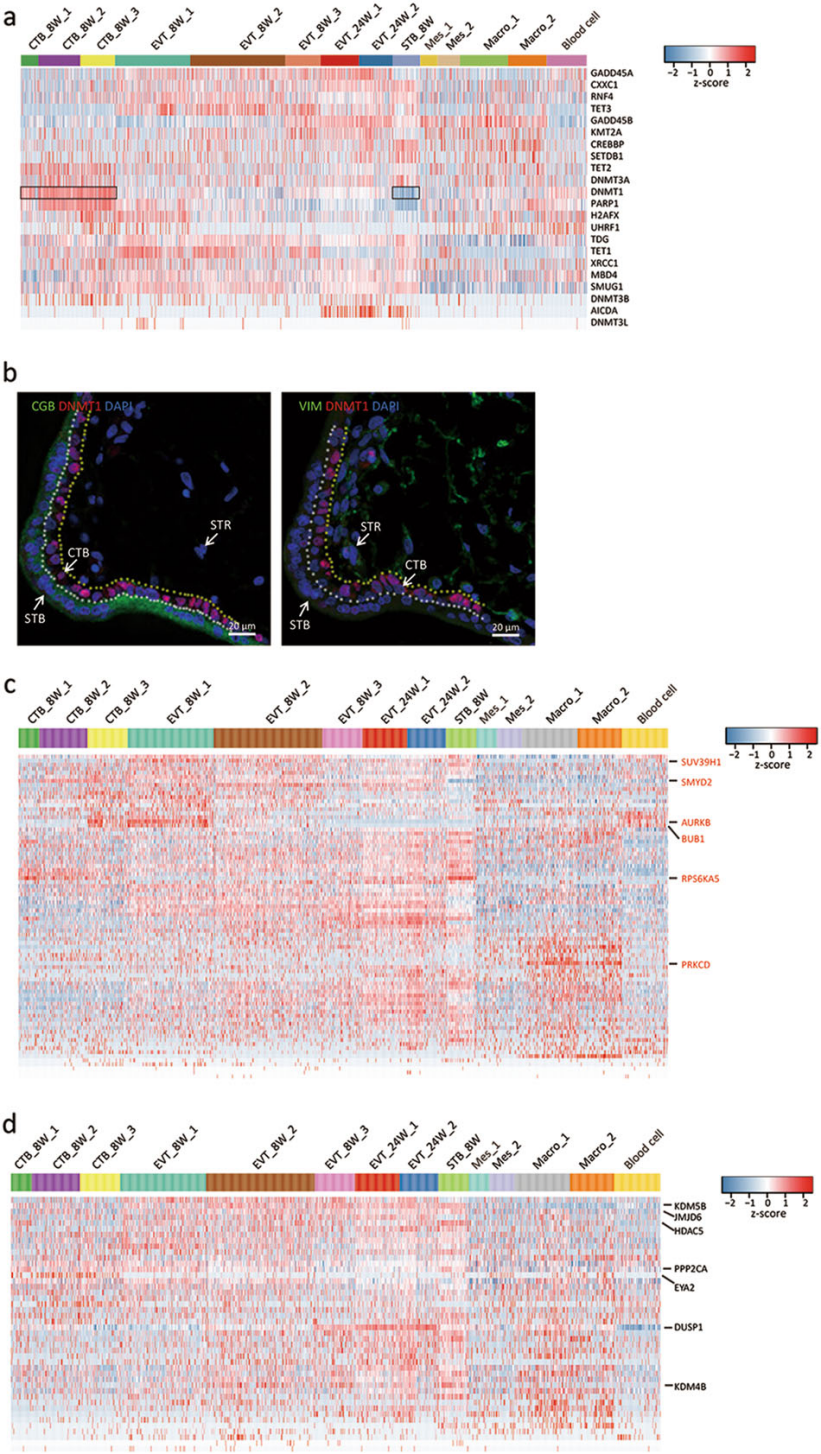
Expression of epigenetic modification-related genes and imprinted genes in the 14 cell subtypes. **a** Heatmap of the genes associated with DNA methylation and demethylation. **b** Immunofluorescence staining of the indicated molecules in the human placental villi at 8 weeks of pregnancy. The cells between the white and yellow dashed lines are CTBs. STR, stromal cells. **c, d** Heatmaps of the chromatin modification writers (**c**) and erasers (**d**) in each cell subtype

Epigenetic modification, which results in cellular differentiation and reprogramming,^34^ plays an important role in maintaining a functional placenta.^35^ To systematically study epigenetic modification during trophoblast development, we analyzed the expression profiles of genes whose products participate in DNA methylation and chromatin modification in the 14 subtypes of cells (Fig. 5a). DNMT1, which functions to maintain DNA methylation patterns during cellular replication, was highly expressed in all CTB_8W cell subtypes, and the expression pattern of this gene was further confirmed by immunofluorescence staining of 8 week human placental villi (Fig. 5b). DNMT3A, DNMT3B and UHRF1 did not exhibit obvious expression variations among the 14 subtypes of cells (Fig. 5a). Of the TET family of DNA dioxygenases,^36^ TET1 and TET3 were highly expressed in all EVT_8W cell subtypes, whereas TET2 was most highly expressed in CTB_8W subtypes. AICDA, a cytidine deaminase involved in DNA demethylation, was only slightly expressed in EVT_24W cells. Many genes encoding chromatin modification writers or erasers also exhibited apparent cell subtype-specific expression patterns (Fig. 5c, d and Supplementary information, Table S3). For example, the writer gene *SUV39H1* and eraser genes, such as *KDM5B*, *JMJD6* and *HDAC5*, were highly expressed in all trophoblast cell subtypes except for the CTB subtypes, whereas *SMYD2* was found to be highly expressed in all trophoblasts except for the STB. *RPS6KA5* was extremely highly expressed in the STB. There were also some differences regarding the expression pattern of these genes among the different EVT_8W subtypes; for example, writer genes, such as *AURKB* and *BUB1*, and eraser genes, such as *PPP2CA*, were highly expressed in EVT_8W_1 subtypes. Previously, we demonstrated that EVT_8W_3 cells exhibit some similarities with EVT_24W subtypes in gene expression, and here, we also found these similarities regarding the expression of *PRKCD*, *KDM4B* and *DUSP1*.

Genomic imprinting characterized by parental-specific monoallelic gene expression has evolved in placental mammals and is vital for both the development of the placenta and its function.^37,38^ Due to the importance of imprinted genes in human placental development, we selected 82 imprinted genes (http://www.geneimprint.com/site/genes-by-species) and analyzed their expression patterns in all the cell subtypes (Supplementary information, Figure S6). We observed unique expression patterns of some of the imprinted genes in certain cell subtypes. For instance, maternally imprinted genes, such as *PHLDA2* and *CDKN1C*, were specifically expressed in all trophoblast cell subtypes except for STB_8W. Regarding the STB_8W, the highly and specifically expressed genes included maternally imprinted genes, such as *ZNF331*, *PHACTR2*, *TFPI2*, *PPP1R9A* and *ANO1*, and the paternally imprinted gene *RHOBTB3*. Paternally imprinted genes, such as *PEG3*, *PEG10*, *MEST* and *AIM1*, were highly expressed in all three CTB subtypes. For the EVT cell subtypes (including EVT_8W and EVT_24W), maternally expressed genes, such as *GRB10*, *ASCL2* and *H19*, and paternally expressed genes, such as *GPR1* and *IGF2*, were highly expressed. For the two subtypes of mesenchymal stromal cells, maternally imprinted genes, such as *KCNQ1OT1*, *PHACTR2* and *MEG3*, and paternally imprinted genes, such as *PLAGL1*, *DLK1* and *MEST1*, were highly expressed in the Mes_2 subtype.

### Different cell subtypes from the human placenta secreted diverse polypeptide hormones

**Fig. 6:**
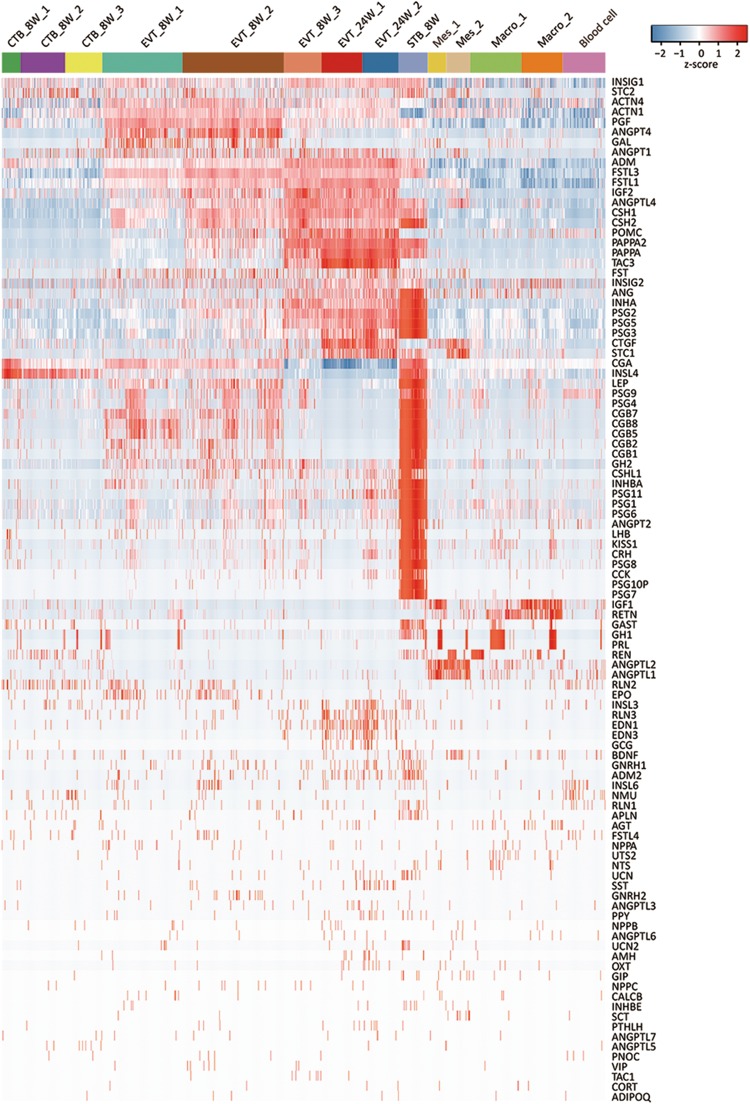
Heatmap of polypeptide hormone genes expressed in the 14 cell subtypes

The human placenta has been identified as the source of many steroid or polypeptide hormones involved in fetal growth and in the maternal adaptation to pregnancy.^6^ The STB has been reported to be the major site of the synthesis and secretion of these hormones.^2,6^ Reports on the secretion of hormones by other cell subtypes, such as CTBs, EVTs and STRs, are rare. To systematically study the production of polypeptide hormones in the 14 subtypes, we analyzed the expression levels of 120 polypeptide hormone genes that have been recognized to be produced by the human placenta based on microarray data in the Gene Expression Omnibus (GEO) and previous reports (Fig. 6).^6,39^ As many as 102 polypeptide hormone genes were detected (Fig. 6 and Supplementary information, Table S4). STB_8W expressed 60 hormone genes, including those of well-documented gene families, such as *CGB* and *PSG*.^39^ To our surprise, the EVT cell subtypes expressed 42 polypeptide hormone genes, among which *CSH1*, *FSTL1*, *PAPPA2*, *TAC3* and several *PSG* genes were highly expressed. Furthermore, the stromal cells also expressed many hormone genes, and the expression levels of *ANGPTL1*, *ANGPTL2*, *ANGPTL4*, *CTGF* and *ACTN1* were high. The spatial and temporal changes in the expression of these polypeptide hormone genes likely reflect different roles for each cell subtype during pregnancy.

## Discussion

**Fig. 7:**
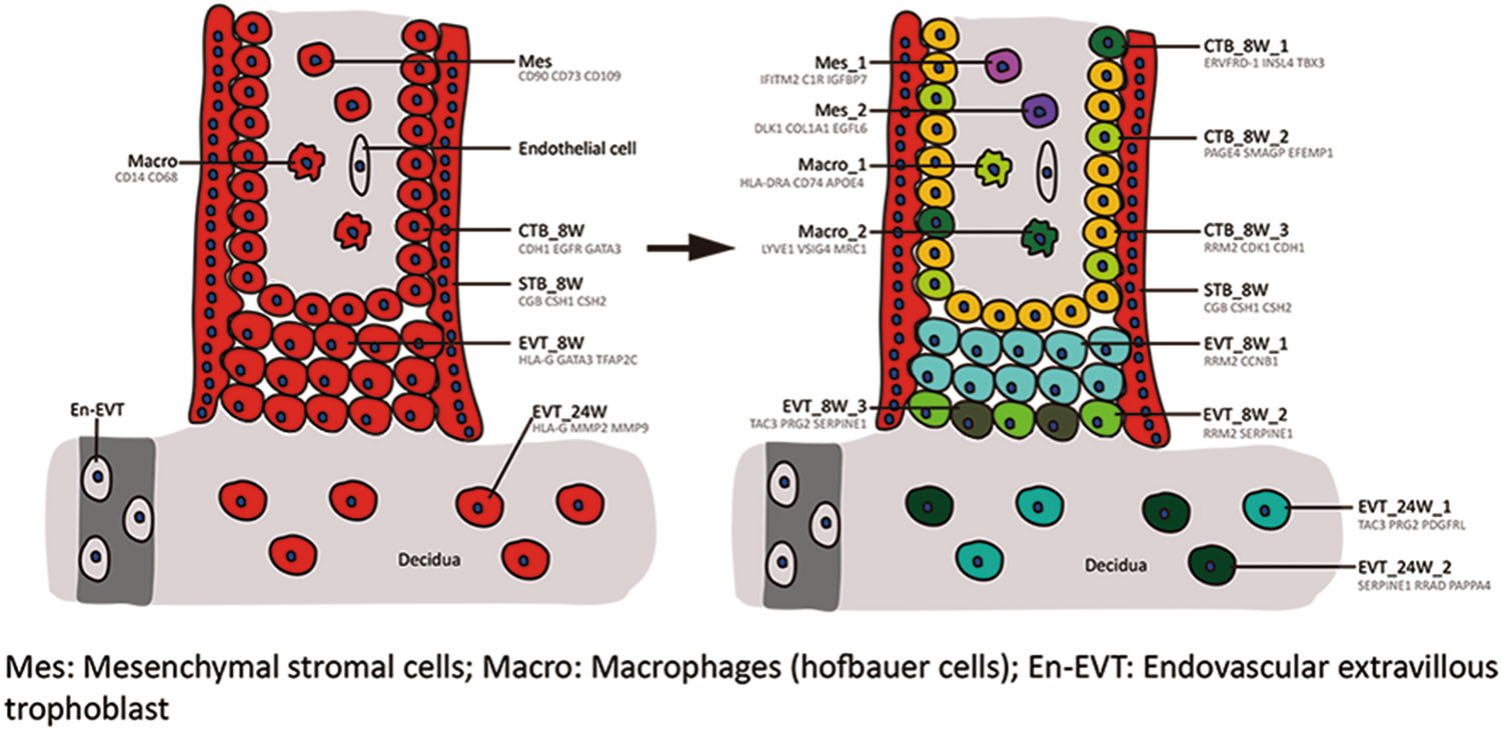
Summary of newly found cell subtypes and their marker genes in the human placenta. Note: the known populations of cells, including endothelial cells and En-EVT, which were not included in this study, are in grey

The human placenta is a highly dynamic and cellularly heterogeneous endocrine organ. This study, focusing on the first and second trimester human placenta, identified new subtypes of placental cells (Figs. 1f and 7): (1) We found three subtypes of CTBs: a proliferative subtype, which may serve as the pool that replenishes the CTB pool; a non-proliferative, Syncytin-2-positive cell subtype, which proved to be the progenitor cells of the STB; and a non-proliferative, Syncytin-2-negative subtype. (2) We found three subtypes of EVTs at 8 weeks of gestation: EVT_8W_1, EVT_8W_2, and EVT_8W_3 subtypes; and two subtypes of EVTs from 24W of gestation: EVT_24W_1 and EVT_24W_2 subtypes. (3) We also identified two subtypes of villous mesenchymal stromal cells and macrophages in the villous stromal core. We have been able to define the cell-type-specific genetic and epigenetic signatures of different cell types as well as their potential functions. We also found that the human placenta expresses many polypeptide hormone genes. In short, this information greatly enriches our knowledge of human placental lineage specification and broadens our understanding of the human placenta as a powerful endocrine organ.

Identifying the stem or progenitor cells for CTBs is crucial for understanding human placental lineage commitment. Two decades ago, mouse trophoblast stem (TS) cells were established from the trophectoderms of mouse blastocysts.^40^ However, it was not until recently that human TS cell lines were derived from the trophectoderm of the human blastocyst and the cytotrophoblast cells from the first trimester human placenta.^41^ CTB_8W_3 cells were highly proliferative compared with the other two CTBs. We believe that these cells are responsible for replenishing the CTBs, and they might serve as trophoblast stem cells. The gene signatures and the functional denotations of this subtype provided in this study may help to simplify the isolation and preparation process and improve the efficiency of the generation of human TS cell lines.

Cell fusion is an essential event during mammalian development that leads to the formation of the zygote, osteoblast, myotube and placental syncytiotrophoblast.^42,43^ Studies of the fusion process during *Drosophila* myotube formation have indicated the existence of a small population of fusion-competent cells. These cells expressed Sticks and Stones (Sns), which interacts with the transmembrane protein Duf on the founder cells to initiate the intracellular signaling required for cell fusion.^44^ In mice, myomaker has been demonstrated to be required for myoblast fusion^45^ and renders cells fusion-competent.^46^ Several reports on human placental trophoblast fusion have also indicated the possible presence of fusion-competent cells,^47,48^ and recently, our work revealed that the G0-restricted expression of Syncytin-2 is a prerequisite for the fusion of CTB into STB.^25^ In this study, we demonstrated, both bioinformatically and experimentally, that the intermediate STB-committed CTB subtype (CTB_8W_1) does exist *in vivo*. The fusion-competent CTBs are found within the CTB layer with no cell column attached. The major characteristics of the placental fusion-competent cells included cell cycle exit, high expression of *Syncytin-2* and adhesion genes, and the initiation of expression of hormone genes, including *INSL4* and *CGA*. Notably, Syncytin-2 is sufficient to reconstitute fusion in nonfusogenic cells. Thus Syncytin-2 alone can make cells competent for fusion and induce fusogenicity. Regarding myomaker, additional proteins, such as myomixer^49^ or myomerger,^46^ are required for the induction of fusion in nonfusogenic fibroblasts. Taken together, these findings highlight Syncytin-2 as a signature for placental fusion-competent cells.

In addition to trophoblast cells, we also found interesting subtypes of cells in the villous stroma. Macro_2 cells in the human placenta strongly expressed Apoe, C1qc and Csf1r (Supplementary information, Table S1). These macrophages were in an activated state and might function to clear the cellular debris during placental development. Interestingly, in a recent study, Han and colleagues used Microwell-Seq to analyze 400,000 cells from most of the major mouse organs and found a similar population of macrophages in the 14.5 d placenta, i.e., Apoe macrophages, which also highly expressed Apoe, C1qc and Csf1r. The existence of this cell subtype in both human and mouse placentas implies its critical role in placental development.

The placenta functions not only as the lung, gut, kidney, pancreas, heart and liver of the fetus during gestation but in a sense as another “brain” in the mother that drives maternal adaptation to fetal development during pregnancy.^6,39^ Maternal adaptations during pregnancy include local angiogenesis and vasodilation in the uterus, shifts of progesterone production from the ovary to the placenta, increases in cardiac output, amongst others. These are achieved through the coordination of the expression and functions of numerous hormones secreted by the placenta.^50–53^ Our study systematically examined the gene expression profiles of 102 peptide hormones in the human placenta. As expected, the STB was a gigantic pool for polypeptide hormone gene expression. Surprisingly, we found that EVTs also expressed many polypeptide hormone genes, which suggests that, in addition to anchoring the fetus to the maternal uterus,^2^ EVTs are also endocrine cells that might facilitate better crosstalk between EVTs and both maternal decidual cells and immune cells to establish a suitable environment for pregnancy. Further investigation of these hormones is required to help us understand the roles they play in fetal growth and maternal adaptations during pregnancy.

In conclusion, our study provides new insights into human placental development and function. Notably, the cell subtypes in the placenta include many more than previously known and they vary with the gestational stage. Because of the variations in the abundance of cells in the placenta and the technical limitations of cell isolation, we did not identify some known cells, such as endothelial cells and endovascular EVTs (Fig. 7). Nonetheless, the data obtained in our study will help us to better understand pregnancy and develop possible therapeutic strategies to improve pregnancy outcomes.

## Materials and methods

### Ethics statement, Informed consent and sample collection

This research was approved by the Ethics Committee of the 306th Hospital of PLA (research license number 201507). Signed informed consent was obtained from all women who donated their placentas. Samples were used according to standard experimental protocols approved by the Ethics Committee of the Institute of Zoology, Chinese Academy of Sciences. Placentas from the first trimester (8 weeks of gestation) and the second trimester (24 weeks of gestation) were collected for the isolation of single cells or for immunohistochemistry and immunofluorescence studies. For the frozen sections, human placental tissues were fixed in 4% paraformaldehyde (PFA, Sigma-Aldrich, St Louis, MO, USA) for 2 h at room temperature and then infiltrated with 10% sucrose for 1.5 h before being frozen, embedded in optimal cutting temperature (OCT, Sakura, Torrance, CA, USA) compound and sectioned. Fifteen-micrometer frozen sections were mounted on 3-aminopropyl-triethoxysilane (APES)-coated slides, air-dried and stored at – 80 °C until use. For paraffin sectioning, placental tissues were fixed in 4% PFA, dehydrated and embedded in paraffin for histological sectioning.

### Isolation of single cells from first- and second-trimester placentas

To isolate the EVT, CTB, STB and STR cells from the first-trimester placentas, villi from 8 week placentas were scraped from the chorionic membrane. Immunohistochemistry staining revealed that no VIM-positive cells were found at the tip of villi, indicating the lack of contamination from maternal tissues, such as the decidua (data not shown). The villi were minced into small pieces with scissors, digested twice with an enzyme cocktail containing 0.125% trypsin (Sigma-Aldrich), 0.05% type IV collagenase (Sigma-Aldrich) and 0.04% DNase (Sigma-Aldrich), and diluted in Dulbecco’s modified eagle medium (DMEM, HyClone, Logan, UT, USA) at 37 °C for 8 min in a shaking incubator. The suspension was filtered through a 70-µm cell strainer, and the enzymatic reaction was stopped by adding 5% fetal bovine serum (FBS, Gibco, Grand Island, NY, USA). The cell suspension was centrifuged at 300 g for 10 min at 4 °C. The cell pellet was resuspended in 1 ml DMEM containing 5% FBS, and the suspension was loaded onto a discontinuous Percoll (GE, Boston, MA, USA) gradient with 7 layers (10, 20, 30, 40, 50, 60 and 70%) and centrifuged at 1200 g for 15 min. The cell fractions on the 30, 40 and 50% interfaces were collected and washed with DMEM. The cell pellets were resuspended in DMEM containing 2% FBS for magnetic activated cell sorting (MACS). Phycoerythrin (PE)-conjugated mouse anti-HLA-G (Sigma-Aldrich) and anti-CDH1 (BD, Franklin Lakes, NJ, USA) antibodies combined with anti-PE microbeads (Miltenyi, Bergisch Gladbach, Germany) were selected for the MACS enrichment of the EVTs, CTBs and STRs from the single-cell suspension according to the manufacturer’s instructions. Briefly, the EVTs were enriched first with anti-HLA-G antibody, and the STBs were purified using a microscope and a mouth pipette from the sorted EVT population based on their sizes. Then, the CTBs were enriched from the remaining cell suspension with anti-CDH1 antibody, and the remaining cells (HLA-G^−^ and CDH1^−^) were mainly STRs. The purity of each population of cells was confirmed by immunofluorescence staining. We designated EVTs, CTBs, STBs and STRs from the 8 week placental villi as EVT_8W, CTB_8W, STB_8W and STR_8W cells, respectively. To isolate EVTs from the second-trimester placenta, the basal plate was dissected from the villi of a 24 week placenta using a dissection microscope and minced into small pieces. The cells were dissociated and obtained according to the procedure described above. The EVTs (HLA-G^+^) were purified from the population with PE-conjugated anti-HLA-G antibody and anti-PE microbeads using MACS according to the manufacturer’s instructions. We designated the EVTs from the 24 W decidua as EVT_24W cells. Altogether, five populations of cells were isolated: EVT_8W, CTB_8W, STB_8W, STR_8W and EVT_24W cells.

### Histology and immunofluorescence

The immunofluorescence and immunohistochemistry stainings were conducted using antibodies directed aginst CK7 (ZSGB, Beijing, China), HLA-G (Sigma-Aldrich), VIM (ZSGB), MRC1 (Abcam, Cambridge, MA, USA), Syncytin-2 (Sigma-Aldrich), CDH1 (Santa Cruz), CD68 (Santa Cruz), CD74 (Santa Cruz), DLK1 (ZEN Bioscience, Chengdu, Sichuan, China), TAC3 (Sigma-Aldrich), and SERPINE1 (ZEN Bioscience). Anti-rabbit Alexa Fluor 555 (Invitrogen, Carlsbad, AR, USA) and anti-mouse Alexa Fluor 488 (Invitrogen) were used as secondary antibodies. For immunofluorescence staining, paraffin sections were cleared of paraffin, hydrated and “epitope-retrieved” in heated citrate buffer as described by Fu et al.^54^ The sections were blocked in 2% bovine serum albumin (BSA, Sigma-Aldrich) for 1 h at room temperature and then incubated with primary antibodies at 4 °C in a humidified chamber overnight, followed by incubation with secondary antibodies for 1 h in the dark at room temperature. The nuclei were counterstained with 4′,6-diamidino-2-phenylindole (DAPI, Sigma-Aldrich) and the slides were mounted in mounting medium and stored at −20 °C in the dark. For immunofluorescence staining, frozen sections were first permeabilized in 1% Triton X-100 for 20 min followed by the primary and secondary antibody staining as described for the immunofluorescence staining of the paraffin sections. Immunohistochemistry was performed with an immunohistochemistry kit (ZSGB) following the manufacturer’s instructions, and the sections were counterstained with haematoxylin. For immunofluorescence staining, the cell suspension was spread on APES-coated slides and air dried. The specimens were fixed in 4% PFA and incubated with the primary and secondary antibodies as described above. Images were captured using a Zeiss LSM 780 confocal microscope (Zeiss, Jena, Thuringia, Germany).

### Live-cell imaging

293 T cells transfected with Syncytin-2/EGFP or Syncytin-2/mCherry expression plasmids separately or together and then subjected to time-lapse microscopy. Cells were imaged using an Eclipse Ti model inverted microscope (Nikon, Tokyo, Japan) with a 20 × objective lens (numerical aperture, 0.95; Nikon), an Orca ER model camera (Hamamatsu, Shizuoka, Japan), and a Perfect Focus System (Nikon). The microscope was surrounded by a custom-made enclosure to maintain a constant temperature (37 °C) and atmosphere (5% CO_2_ and 95% air). The filter sets used were as follows: EGFP (488 nm) and mCherry (561 nm). The laser power was maintained at less than 10%. Image acquisition was controlled using the Volocity software package (Perkin-Elmer, Waltham, MA, USA). The images were recorded every 40 s.

### Single-cell transcriptome library preparation and sequencing

Cells were resuspended in 1% BSA/PBS. Single cells with a volume less than 0.1 μl were deposited with a mouth pipette into 2.5 µl lysis buffer and subsequently reverse transcribed and amplified using the modified smart-seq2 method.^55^ We added barcodes and unique molecular identifiers (UMIs) appropriate for highly parallel 3′ end RNA sequencing. As many as 88 cells were pooled together after amplification and purified through two rounds using Ampure XP beads (Beckman, Brea, CA, USA). These pooled cells were used to construct a single library that was sequenced on an Illumina Hiseq4000 with 150-bp paired-end reads.

### Single-cell RNA-seq data pre-processing and quality control

After obtaining the paired-end reads for a library, read 2 was used to obtain the cell barcodes in the library to further split the reads according to their cell (barcode) IDs, and the UMI sequences were simultaneously recorded. Then, read 1 was selected for each cell, and the raw reads were trimmed to remove any TSO or poly (A) sequence. Quality control was subsequently performed on these trimmed reads to eliminate adapter contamination and low-quality bases. Next, the cleaned read 1 sequence for each cell was aligned to the human genome (hg19) using TopHat (version 2.0.14)^56^ with the default settings, and uniquely mapped reads were kept. HTSeq^57^ was used to count the uniquely mapped reads on each gene, and the duplicated UMI sequences inside each gene were removed to estimate the abundances of the gene transcripts. The expression level (RefSeq annotation) of a gene was normalized into transcripts-per-million mapped reads (TPM), which was calculated as the total UMI for a gene divided by the sum of the UMIs from each gene in a given cell multiplied by 1 000 000.

We obtained 1 million mapped reads on average for each cell. Stringent criteria were used to filter out low-quality cells: First, high-quality cells were required to be detected with at least 3 000 genes. Second, the Pearson correlation for every pair of cells was calculated, and the second maximum pair-wise correlation was required to be greater than 0.6. Third, outlier cells identified by t-SNE and PCA analyses were removed. In this manner, 1471 of the 1567 cells examined were qualified for further analysis. In total, 16 845 of 24 153 genes with TPMs over 5 in at least 2 cells were used for analysis.

### Clustering analysis on all cells

We combined several methods for cell sample clustering. The dissimilarity matrix was built by calculating (1-β)/2, where β is the Spearman correlation of each cell pair. Hierarchical clustering was performed on the dissimilarity matrix using the ‘hclust’ R function with the ‘average’ method, and the clusters were identified using the R function ‘cutreeDynamic’ with the ‘hybrid’ method.^58^ The clusters and cell types were visualized via t-distributed stochastic neighbor embedding (t-SNE, as implemented in the’Rtsne’ package) on the dissimilarity matrix. PCA was performed using the Seurat package with highly variant genes.^59^

### Cell cycle analysis

Cell cycle-associated genes identified in a previous study^60^ were divided into two classes based on involvement in the G1/S or G2/M phases. We calculated the average expression of these cell cycle-associated genes, and only cells with average expression of at least 100 were defined as cycling cells. The ratios of cycling cells were calculated as the percentage of cycling cells in each cell subtype.

### DEG analysis

Cell type-specific genes were identified by running Seurat with the ‘find_all_markers’ function on a log-transformed expression matrix. Marker genes between two cell types were found using the Seurat ‘find.markers’ function. Heatmaps, box plots, dot plots and violin plots were created using R. GO analysis was performed via the DAVID website.^61^

### Pseudotime analysis

The Monocle2 packages for R were used to determine the differentiation pseudotimes of the different cell subtypes. Genes with high dispersion (more than twice the fitted dispersion) were selected for unsupervised ordering of the cells.

### Transcription master regulator analysis

The transcriptional regulatory networks of given cell types were first built using the ARACNe-AP software package.^62^ In total, 1 568 human transcription factors from Animal TFDB were used in the analysis.^63^ We analyzed two differentiation routes, i.e., cytotrophoblasts to extravillous trophoblasts and cytotrophoblasts to syncytiotrophoblast. Then, master regulator analysis was performed on each differentiation route using the MARINa implementation of the ssmarina package.^64^

### Analysis of imprinted genes and hormone genes

Imprinted genes were downloaded from the geneimprint website (http://www.geneimprint.com/site/genes-by-species), and only genes with ‘Imprinted’ status and genes detected with a TPM over 2 in at least 30 cells were used for further analysis. Hormone genes were collected from the Gene Expression Omnibus and published reports, and genes detected with a TPM over 2 in at least 30 cells were used for further clustering analysis.

### GSEA analysis

We used Gene Set Enrichment Analysis with default parameters (http://software.broadinstitute.org/gsea/index.jsp) to identify gene sets that exhibited significant and concordant differences between two given biological statuses.^65^ The a priori defined sets of genes we used were from KEGG pathways (c2.cp.kegg, v5.2.symbols.gmt).

### Data access

Our data have been deposited in the NCBI Gene Expression Omnibus (GEO) under accession number GSE89497.

## Electronic supplementary material

Supplementary information, Table S3

Supplementary information, Movie S1

Supplementary information, Movie S2

Supplementary information, Figure S1

Supplementary information, Figure S2

Supplementary information, Figure S3

Supplementary information, Figure S4

Supplementary information, Figure S5

Supplementary information, Figure S6

Supplementary information, Table S1

Supplementary information, Table S2

Supplementary information, Table S4

Supplementary information, Movie legend
