# Supplementary material for: Single-cell RNA-seq reveals the diversity of trophoblast subtypes and patterns of differentiation in the human placenta
Source: Cell Res. 2018 Jul 24;28(8):819–32. doi: 10.1038/s41422-018-0066-y (PMC6082907; doi:10.1038/s41422-018-0066-y)
Supplement: Supplementary file 4 — Supplementary information, Figure S1 [file 41422_2018_66_MOESM4_ESM.pdf]

Supplementary Figures and Legends

Figure S1

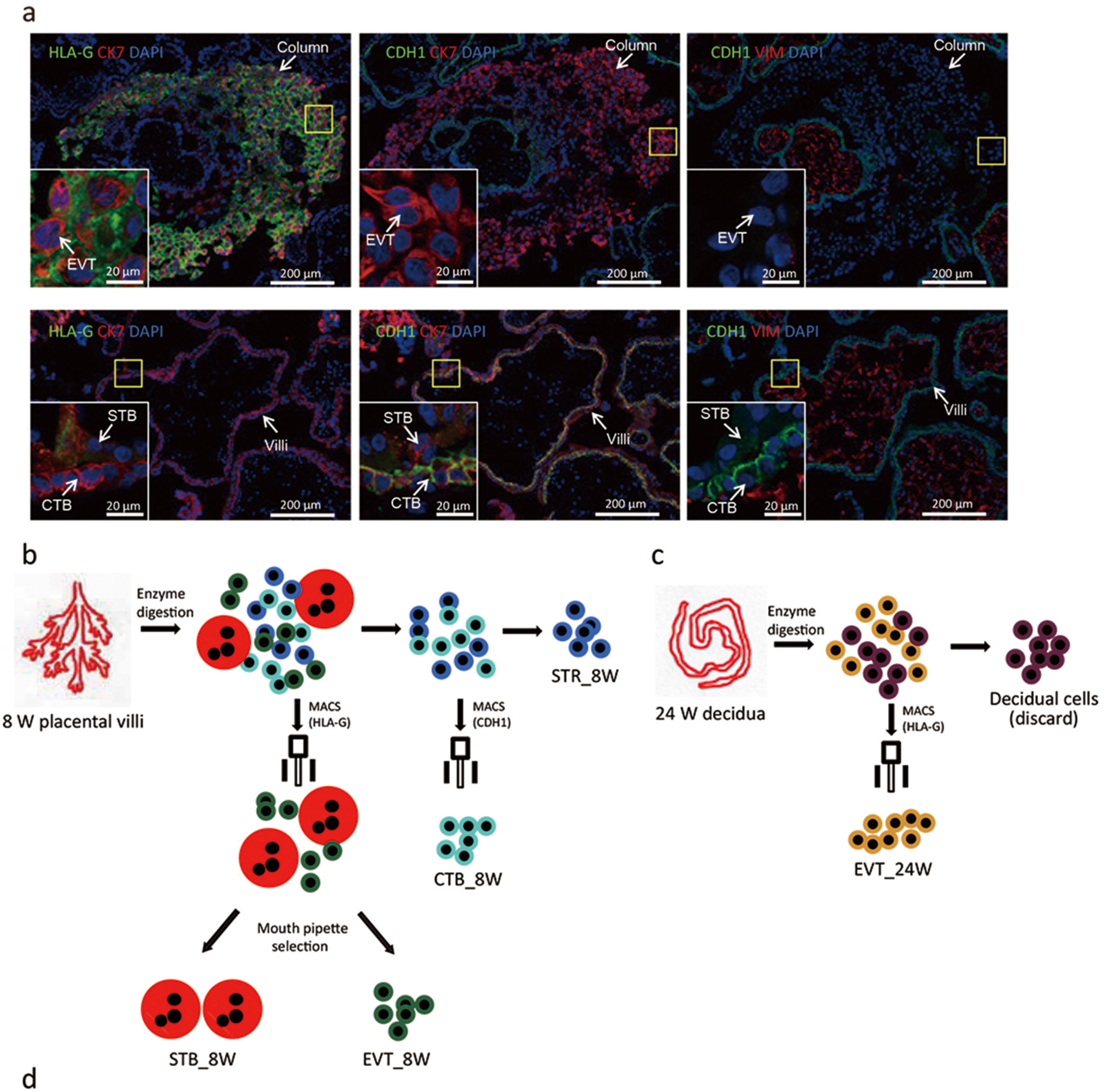

d

Cell Type	Embryo#1	Embryo#2	Embryo#3	Embryo#4	Embryo#5	Embryo#6	Embryo#7	Embryo#8	Total cell number
STR_8W	88					95	240	192	
CTB_8W	224			24					
EVT_8W	88		64			96	96	96	
STB_8W		32	32						
EVT_24W					200				1,567

Figure S1. Strategies for sorting different cell populations from 8 W villi and 24 W decidua in the human placenta.
a Immunofluorescent staining of the column (upper panel) and villi (lower panel) from the 8W human placenta using indicated antibodies. Cytokeratin 7 (CK7): a marker for epithelial trophoblasts, including cytotrophoblast cells (CTBs), extravillous trophoblast cells (EVTs) and syncytiotrophoblast (STB). Vimentin (VIM): a marker for stromal cells. White-framed areas are the magnifications of the corresponding yellow-framed areas within the same image.
b, **c** Methods for sorting different cell populations by MACS from 8W villi and 24W decidua of the human placenta. **d** Sample information for the cells harvested for single-cell RNA-seq.
