# Supplementary material for: Single-cell RNA-seq reveals the diversity of trophoblast subtypes and patterns of differentiation in the human placenta
Source: Cell Res. 2018 Jul 24;28(8):819–32. doi: 10.1038/s41422-018-0066-y (PMC6082907; doi:10.1038/s41422-018-0066-y)
Supplement: Supplementary file 5 — Supplementary information, Figure S2 [file 41422_2018_66_MOESM5_ESM.pdf]

Figure S2

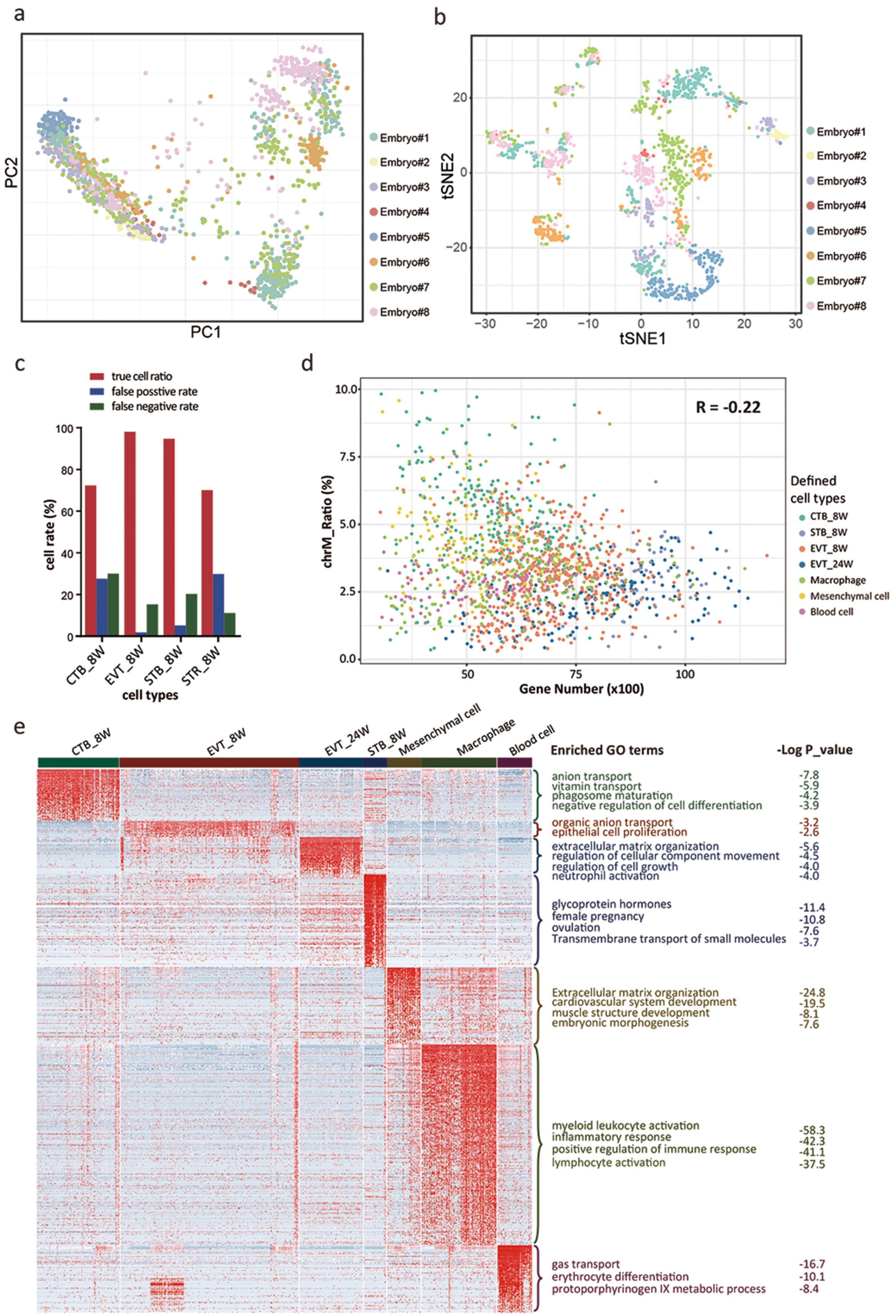

Figure S2. Single-cell transcriptome profiles distinguish the 7 known types of cells in the human placenta.
a PCA analysis of the cells from 8 human embyos. **b** T-SNE plot showing the cells from 8 human embyos.
c Cell purity of different cell types in each population based on the transcriptome analysis of each cell.
d Scatter plot showing the correlation between expressed gene number and mitochondria mapped reads for each cell. **e** GO analysis of the 7 defined cell types from human placenta with different expressed genes (DEGs).
