# Supplementary material for: Single-cell RNA-seq reveals the diversity of trophoblast subtypes and patterns of differentiation in the human placenta
Source: Cell Res. 2018 Jul 24;28(8):819–32. doi: 10.1038/s41422-018-0066-y (PMC6082907; doi:10.1038/s41422-018-0066-y)
Supplement: Supplementary file 6 — Supplementary information, Figure S3 [file 41422_2018_66_MOESM6_ESM.pdf]

Figure S3

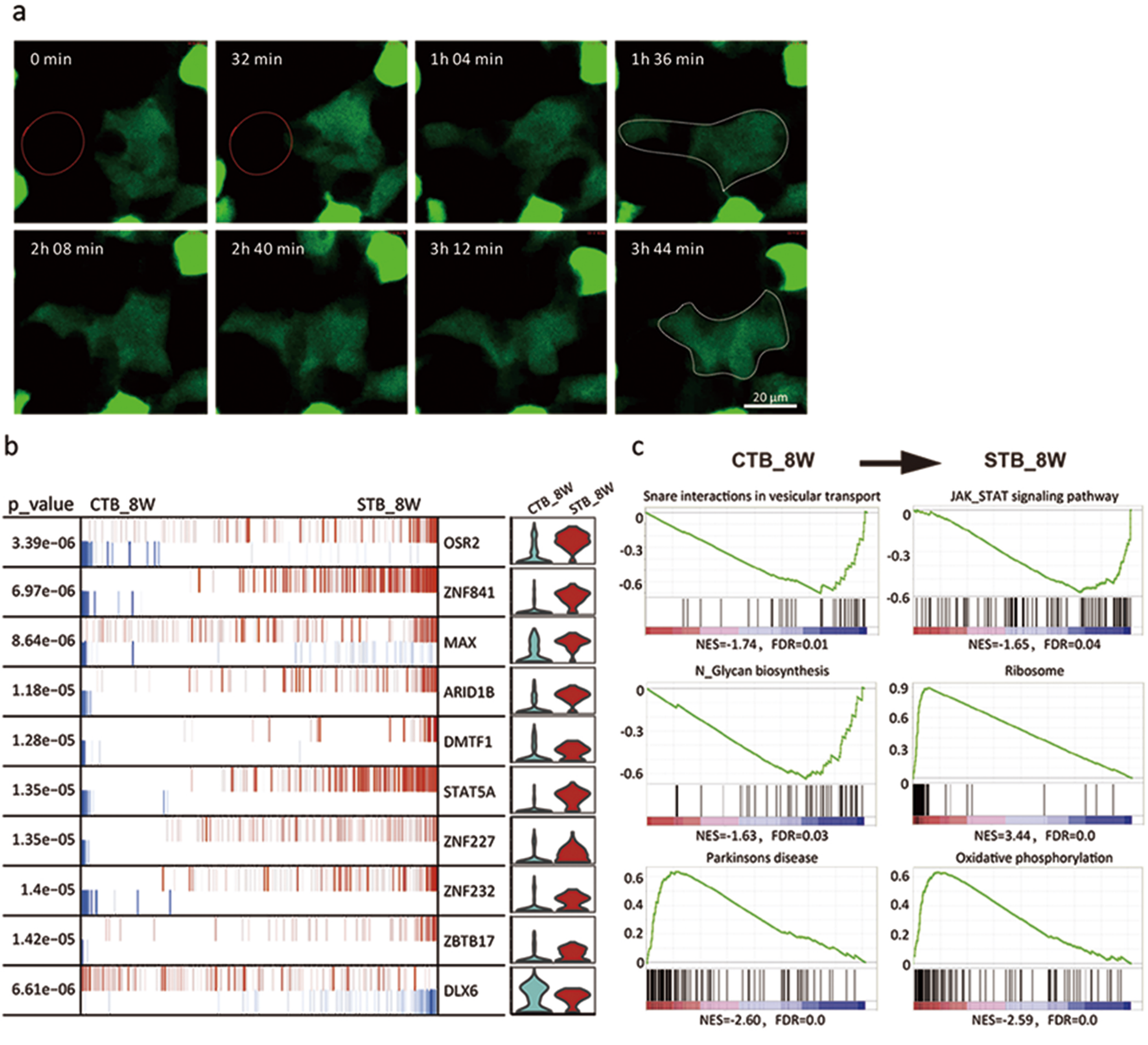

Figure S3. Verification of fusion-competent cells.

a Representative still images for movie S2, a time-lapse imaging of the fusion process of 293T cells with or without (red circled dashed line) overexpression of Syncytin-2. White circled dashed line indicates the fused cell. **b** Top 10 transcription factors regulating the indicated cell subtype transition. **c** GSEA enriched signaling pathways and their regulation patterns in the transition between indicated cell subtypes.
