# Supplementary material for: Single-cell RNA-seq reveals the diversity of trophoblast subtypes and patterns of differentiation in the human placenta
Source: Cell Res. 2018 Jul 24;28(8):819–32. doi: 10.1038/s41422-018-0066-y (PMC6082907; doi:10.1038/s41422-018-0066-y)
Supplement: Supplementary file 7 — Supplementary information, Figure S4 [file 41422_2018_66_MOESM7_ESM.pdf]

Figure S4

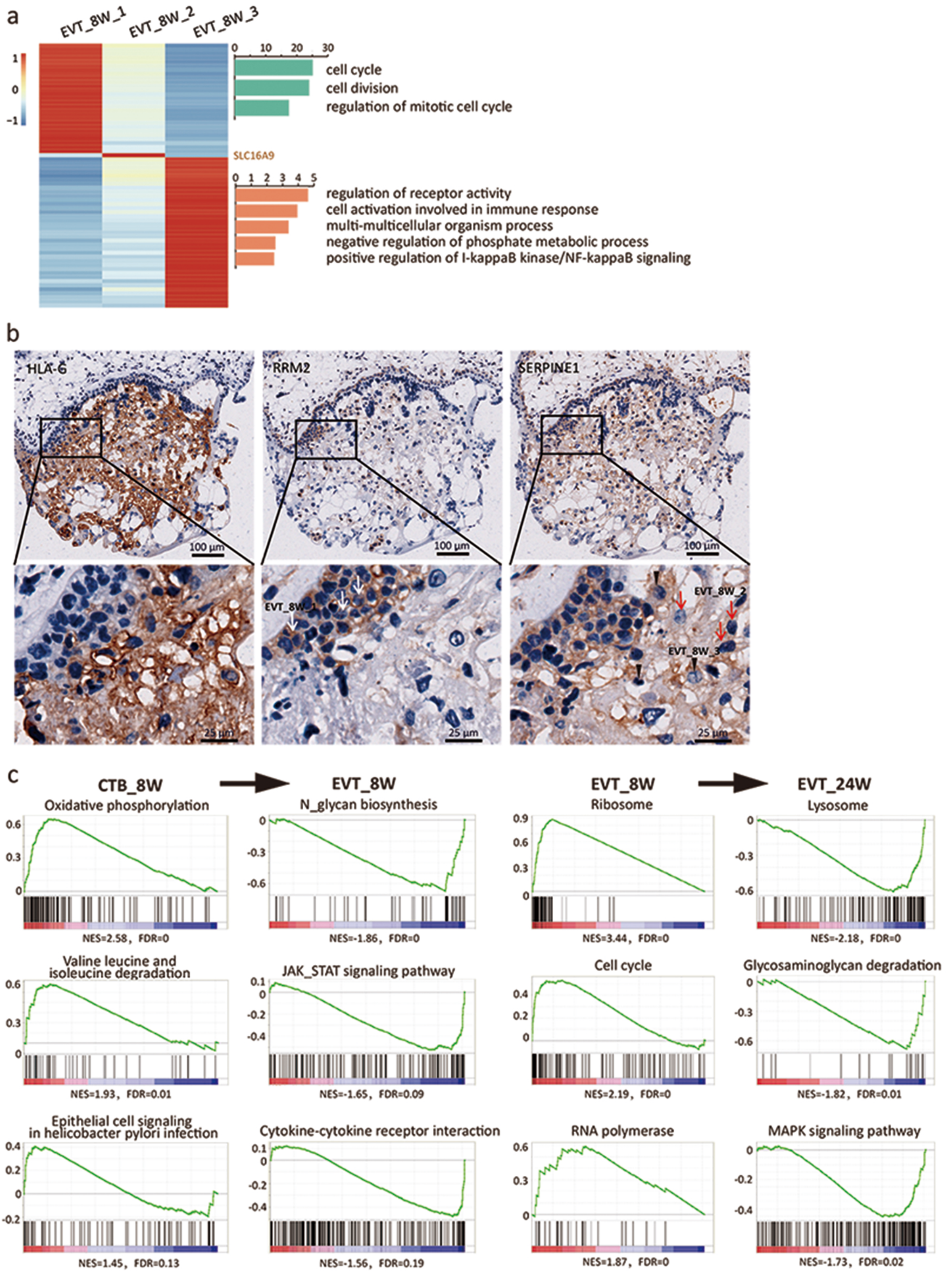

Figure S4. EVT subtypes and differentiation of CTBs into EVTs at the first and second trimesters.
a GO analysis of DEGs for the three subtypes of EVT_8W cells. **b** IHC using indicated antibodies on serial sections of 8W human placental villi. White arrows, EVT_8W_1 cells; red arows, EVT_8W_2 cells; arrowheads, EVT_8W_3. **c** GSEA enriched signaling pathways and their regulation patterns in the indicated transition.
