# Supplementary material for: Single-cell RNA-seq reveals the diversity of trophoblast subtypes and patterns of differentiation in the human placenta
Source: Cell Res. 2018 Jul 24;28(8):819–32. doi: 10.1038/s41422-018-0066-y (PMC6082907; doi:10.1038/s41422-018-0066-y)
Supplement: Supplementary file 8 — Supplementary information, Figure S5 [file 41422_2018_66_MOESM8_ESM.pdf]

# Figure S5

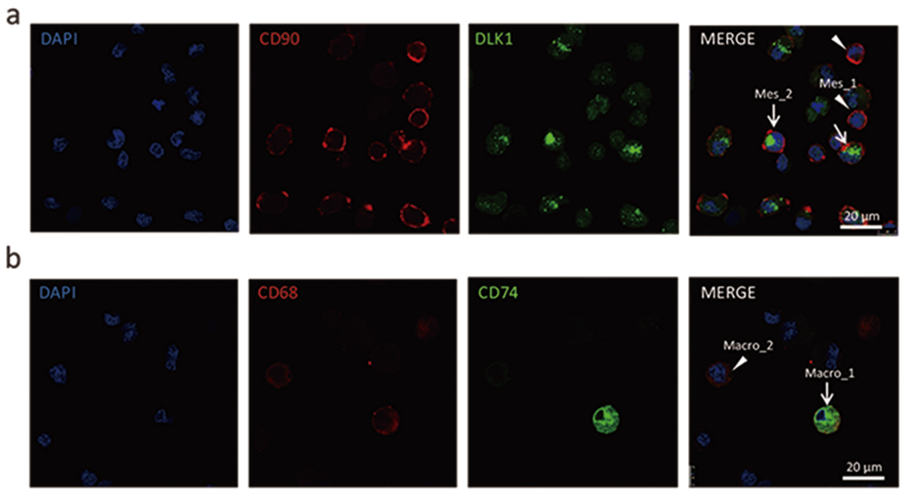


**Figure S5. Verification of distinct cell subtypes from the first trimester villous stromal core**

**a** Representative images showing immunofluorescence staining of DLK1 in the stromal cells sorted with CD90 antibody. Arrows and arrowhead indicate stromal cells with high and low expression of DLK1, respectively. **b** Representative images showing immunofluorescence staining of CD74 of cells in the stroma sorted with CD68 antibody. The arrow and arrowhead indicate macrophages with high and low expression of CD74, respectively.
