# Supplementary material for: Single-cell RNA-seq reveals the diversity of trophoblast subtypes and patterns of differentiation in the human placenta
Source: Cell Res. 2018 Jul 24;28(8):819–32. doi: 10.1038/s41422-018-0066-y (PMC6082907; doi:10.1038/s41422-018-0066-y)
Supplement: Supplementary file 9 — Supplementary information, Figure S6 [file 41422_2018_66_MOESM9_ESM.pdf]

Figure S6

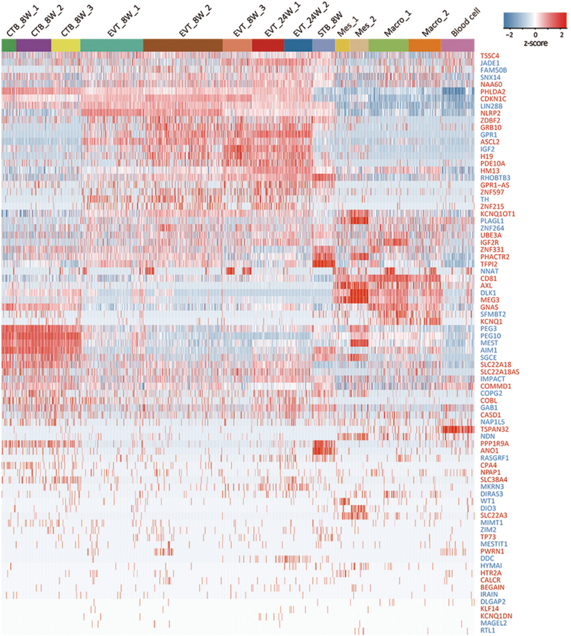

Figure S6 Heatmap of imprinted genes in 14 different cell subtypes from human placenta
Red, maternally expressed genes; blue, paternally expressed genes.
