# Supplementary material for: Single-cell RNA-seq reveals the diversity of trophoblast subtypes and patterns of differentiation in the human placenta
Source: Cell Res. 2018 Jul 24;28(8):819–32. doi: 10.1038/s41422-018-0066-y (PMC6082907; doi:10.1038/s41422-018-0066-y)
Supplement: Supplementary file 13 — Supplementary information, Movie legend [file 41422_2018_66_MOESM13_ESM.docx]

**Supplementary Movies**

**Movie S1. Time-lapse imaging of 293T cells showing cell fusion between Syncytin-2 overexpressing cells (see also the images shown in Figure 2g).**

**Movie S2. Time-lapse imaging of 293T cells showing cell fusion between Syncytin-2 overexpressing and Syncytin-2 non-expressing cells (see also the images shown in Supplementary information, Figure S3a).**
